# Plant-based diets and total and cause-specific mortality: a meta-analysis of prospective studies

**DOI:** 10.3389/fnut.2025.1518519

**Published:** 2025-01-20

**Authors:** Qiwang Mo, Jizhongrong Wu, Yi Lu, Xiao Zhang

**Affiliations:** Department of Urology, Shengzhou People’s Hospital (Shengzhou Branch of the First Affiliated Hospital of Zhejiang University School of Medicine, The Shengzhou People’s Hospital of Shaoxing University), Shengzhou, Zhejiang, China

**Keywords:** plant-based diet, mortality, cardiovascular disease, cancer, cohort, meta-analysis

## Abstract

**Objective:**

The adherence to plant-based diets has been shown to positively impact longevity by reducing the incidence and severity of lifestyle-related diseases. Previous studies on the association of plant-based dietary pattern, as evaluated by plant-based dietary index (PDI), healthy plant-based dietary index (hPDI) and unhealthy plant-based dietary index (uPDI), with mortality risk have reported inconsistent results. We performed the present meta-analysis to summarize evidence on this association and to quantify the potential dose–response relationship based on all available cohort studies.

**Methods:**

A comprehensive literature search and systematic review of relevant articles up to October 2024 was performed in PubMed and Scopus. The summary risk estimates (RR) with 95% confidence interval (CI) for the highest versus the lowest category of PDIs, hPDIs and uPDIs were calculated. Dose–response meta-analysis was also performed for studies reporting categorical risk estimates for at least three quantitative levels of PDIs, hPDIs and uPDIs.

**Results:**

A total of 11 eligible cohort studies (13 datasets) were eventually included in this meta-analysis. Participants in the highest quintile of both the PDI and hPDI had a significantly decreased risk of all-cause mortality (pooled HR_PDI_ = 0.85; 95% CI: 0.80–0.90; pooled HR_hPDI_ = 0.86; 95% CI: 0.81–0.92) compared to participants in the lowest quintile. In contrast, the highest uPDI was associated with an increased risk of mortality (pooled HR_uPDI_ = 1.20; 95% CI: 1.11–1.31). Dose–response meta-analysis showed that there was a non-linear relationship between PDI or hPDI level and all-cause mortality (*p* = 0.001 and *p* < 0.001 for non-linearity, respectively). No evidence of a non-linear relationship was observed for uPDI (*p* = 0.596 for non-linearity).

**Conclusion:**

Greater adherence to PDI or hPDI dietary pattern was associated with a lower risk of mortality, whereas uPDI dietary pattern was positively associated with mortality risk. Thus, promoting a plant-based dietary pattern may be a key strategy in improving public health and reducing the burden of diet-related mortality.

## Introduction

In recent years, life expectancy has generally increased worldwide, driven by advances in healthcare, improved living conditions, and vaccination efforts. However, significant disparities persist between high-income and low-income regions (1). While non-communicable diseases (NCDs) such as cardiovascular disease (CVD), cancer, and diabetes are the leading causes of death globally, low-income countries still face high mortality rates due to infectious diseases, malnutrition, and inadequate healthcare infrastructure (2, 3). Additionally, emerging challenges, including climate change, pandemics, and an aging population, continue to influence mortality patterns and present ongoing public health concerns (4–6).

Dietary patterns play a critical role in determining mortality risk, influencing both all-cause and cause-specific outcomes (7). Understanding the long-term effects of various dietary patterns on health outcomes is essential for developing effective nutritional guidelines that promote public health and reduce preventable mortality. Diets rich in fruits, vegetables, whole grains, and nuts have been consistently associated with reduced risks of CVD, cancer, and other NCDs, thereby contributing to lower mortality rates (8). Conversely, dietary patterns high in processed foods, red and processed meats, added sugars, and unhealthy fats are linked to increased morbidity and mortality due to their association with obesity, hypertension, dyslipidemia, and chronic inflammation (9).

The adherence to plant-based diets or those emphasizing nutrient-dense, minimally processed foods has been shown to positively impact longevity by reducing the incidence of lifestyle-related diseases (9). In recognition of its significance, plant-based dietary indexes (PDIs) were introduced in 2016 as a tool to link a plant-based dietary pattern to health outcomes (10). These indexes use a graded scoring system for various food items and include three categories: (1) plant-based diet index (PDI), which assesses the consumption of plant-based foods while reducing intake of animal-based foods; (2) healthful plant-based diet index (hPDI), which assigns positive scores to nutritious plant foods (like whole grains, fruits, vegetables, nuts, and legumes) and negative scores to less healthy plant foods (such as refined grains and potatoes), as well as to animal-based foods; (3) unhealthful plant-based diet index (uPDI), which gives positive scores to less healthy plant foods and negative scores to healthy plant foods and animal foods. Recently, various studies (11–14) have attempted to assess the associations between PDIs and mortality risk with inconclusive results. Due to this inconstancy, our objective was to conduct a systematic review and meta-analysis of prospective studies to investigate whether adherence to the PDIs is associated with the risk of mortality.

## Methods

### Publication search

A comprehensive literature search was conducted in the PubMed and Scopus databases for articles published up to October 2024. The search algorithm included terms such as (“plant-based diet” or “plant based diet”) and (“mortality” or “survival” or “death”). Relevant publications were initially screened based on their titles and abstracts, and all studies meeting the eligibility criteria were retrieved. Reference lists from selected articles and reviews were also checked to identify additional relevant studies. No language restrictions were applied. This systematic review and meta-analysis was conducted following established quality standards for reporting meta-analyses (15, 16).

### Study selection

The included studies met the following criteria: (*i*) the primary exposure was the PDIs (i.e., PDI, hPDI and uPDI); (*ii*) the outcome of interest was all-cause and cause-specific mortality; (*iii*) the study used a prospective cohort design; and (*iv*) relative risk (RRs) with corresponding 95% confidence intervals (CIs) were reported or could be calculated. Studies were excluded if they were reviews, meta-analyses, case reports, or non-human studies. Additionally, studies focusing on other exposures or diseases were not considered. If multiple studies reported data based on the same population, the publication with the largest sample size and longest follow-up was included in our meta-analysis.

### Data extraction

Data extraction was conducted independently by two authors (Q.M. and X.Z.) using a predefined extraction form. From each study, the following information was collected: first author’s name, publication year, study location, study name, study population, sample size (number of participants and cases), participants’ age, dietary assessment, adjusted effect estimates for all exposure categories, and covariates considered in study design or data analysis.

### Quality assessment

The quality of each study was independently evaluated by two authors (Q.M. and X.Z.) using the Newcastle-Ottawa Scale (NOS)^1^. Any disagreements were resolved through a joint review with a third author. The NOS assigns up to nine points per study, with scores of less than 8 indicating lower quality and scores of 8 or higher indicating high quality.

### Statistical methods

The main outcome of our study was all-cause mortality. We also included information on CVD and cancer, which were the leading causes of death worldwide. The strength of the relationship between PDIs and mortality risk was measured using a pooled adjusted RR and its 95% CI estimated by a DerSimonian and Laird random effects model (17). Comparisons were made between the highest and lowest PDI score categories to assess the all-cause and cause-specific mortality risk.

A dose–response meta-analysis followed the methods of Greenland and Longnecker (18) and Orsini et al. (19). We included studies that provided at least three quantitative categories with the number of cases and person-years for each. For studies that only reported overall person-years, we estimated the distribution using the approach of Aune et al. (20). Median or midpoint values for each category were used to represent the dose. If the upper boundary of the highest category was unavailable, we estimated it based on the nearest category. To explore a potential non-linear dose–response relationship between PDIs and mortality, we used restricted cubic splines, with knots placed at the 25th, 50th, and 75th percentiles of the distribution (21). Non-linearity was tested by checking if the second spline’s coefficient was zero.

We assessed study heterogeneity using the *Q* statistic and the *I*^2^ score (22), with heterogeneity defined as low (*I*^2^ < 25%), moderate (*I*^2^ = 25–50%), or high (*I*^2^ > 50%). Meta-regression analysis explored possible sources of heterogeneity, and subgroup analyses were performed based on study region, sample size, and follow-up duration. Sensitivity analysis involved repeating the meta-analysis, excluding each study one by one. Potential publication bias was checked using Begg’s test (rank correlation method) (23) and Egger’s test (linear regression method) (24). All statistical analyses were conducted in STATA 12.0 (StataCorp, College Station, TX), with two-sided *p*-values.

## Results

### Literature search and study characteristics

**Figure 1:**
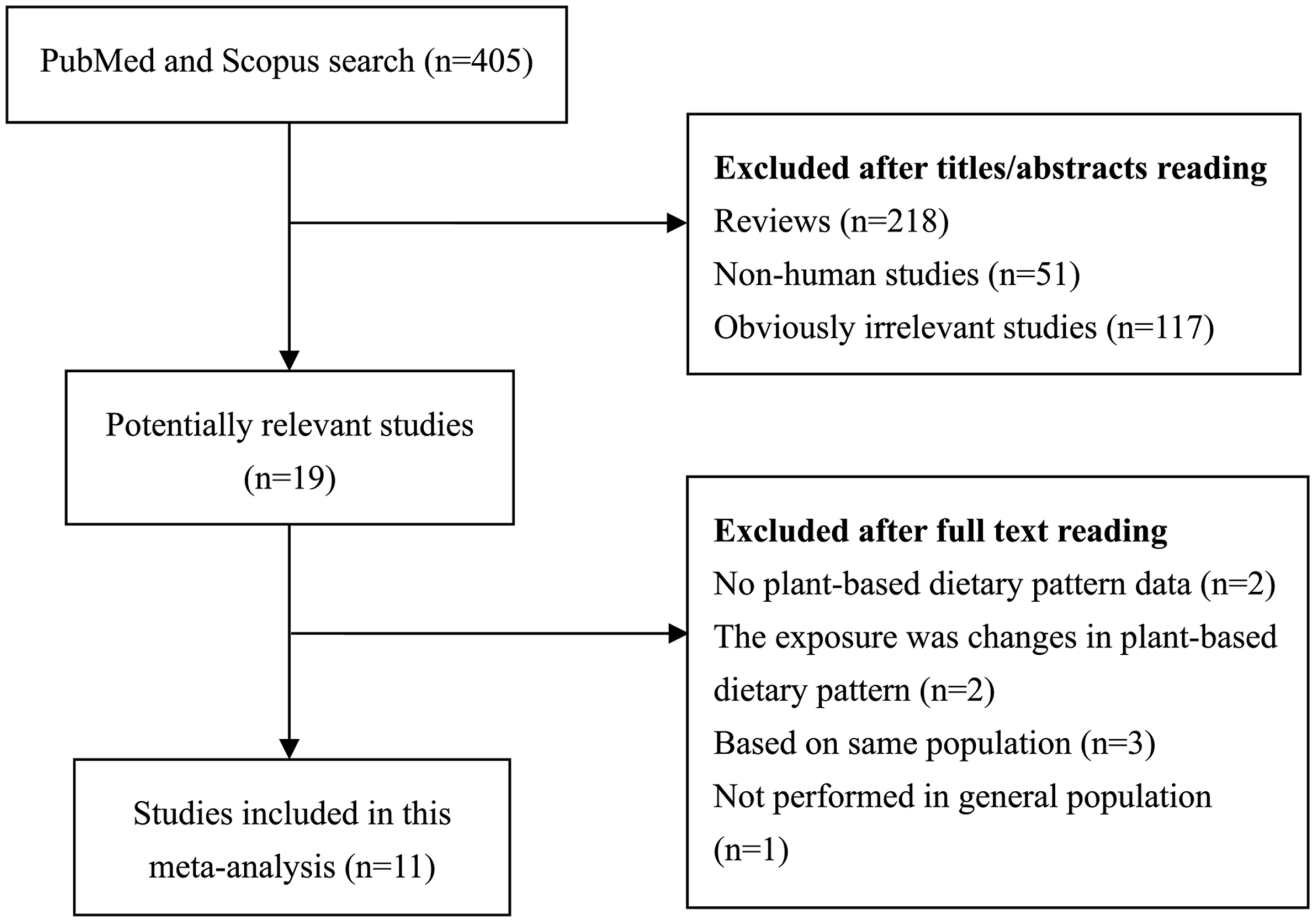
Literature search and study selection.

Figure 1 presents the detailed process of literature review. A total of 11 eligible cohort studies (13 datasets) (11–14, 25–31) were eventually included in this meta-analysis aimed to comprehensively evaluate the relationship between PDIs and mortality risk. These studies were performed in Asia (*n* = 3), North America (*n* = 6), and Europe (*n* = 2). 977,763 participants were included in these studies published between 2019 and 2024. There were 184,160 death events, which was mainly confirmed by linking to National Death Index. Table 1 summaries the main characteristics of all included studies analyzed in this meta-analysis. NOS scores ranged from 7 to 9, with a median value of 8 (Supplementary Table S1).

**Table 1:** Main characteristics of the studies included in the meta-analysis.

Study	Year	Country	Study name	Cases	Cohort	Age, y	Follow-up, y	NOS score
Chen et al. (12)	2024	China	CHNS	792	9,364	54.5 (9.4)	9.2	7
Kim et al. (30)	2024	US	MCS (men)	33,304	66,357	59.0 (8.7)	21.3	8
Kim et al. (30)	2024	US	MCS (women)	31,783	78,372	58.8 (8.8)	21.3	8
Delgado-Velandia et al. (27)	2024	Spain	ENRICA	699	11,825	NA	10.9	8
Zhou et al. (11)	2024	UK	UK Biobank	9,335	189,003	56.0 (8.0)	9.6	7
Shan et al. (29)	2023	US	NHS	31,263	75,230	50.2 (7.2)	36	8
Shan et al. (29)	2023	US	HPFS	22,900	44,085	53.3 (9.6)	34	8
Chen et al. (26)	2022	China	CLHLS	8,937	13,154	86.9 (11.4)	5.7	7
Weston et al. (13)	2022	US	Jackson Heart Study	597	3,635	21–95	15	9
Wang et al. (31)	2022	US	Million Veteran Program	31,136	315,919	65.5 (19–104)	4	7
Li et al. (28)	2022	US	NHANES	4,904	40,074	47.3 (19.4)	7.8	8
Kim et al. (25)	2021	South Korea	ARIC	3,074	118,577	52.7 (8.2)	10	8
Kim et al. (14)	2019	US	NA	5,436	12,168	45–64	25	9

y, year; NOS, Newcastle-Ottawa scale; NA, not available; CHNS, China Health and Nutrition Survey; MCS, Multiethnic Cohort Study; NHS, Nurses’ Health Study; HPFS, Health Professionals Follow-up Study; CLHLS, Chinese Longitudinal Healthy Longevity Survey; NHANES, National Health and Nutrition Examination Survey; ARIC, Atherosclerosis Risk in Communities.

### Main analyses

**Figure 2:**
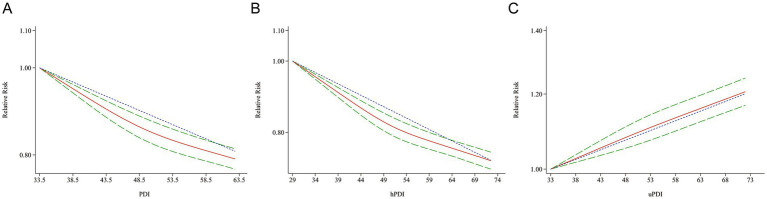
Risk of PDI **(A)**, hPDI **(B)**, and uPDI **(C)** associated with all-cause mortality. Weights are from random effects analysis. RR, relative risk; CI, confidence interval.

Participants in the highest quintile of both the PDI and hPDI had a significantly decreased risk of all-cause mortality (Figure 2, pooled HR_PDI_ = 0.85; 95% CI: 0.80–0.90; pooled HR_hPDI_ = 0.86; 95% CI: 0.81–0.92) compared to participants in the lowest quintile. In contrast, the highest uPDI was associated with an increased risk of mortality (pooled HR_uPDI_ = 1.20; 95% CI: 1.11–1.31). In terms of the cause-specific mortality, comparing the highest versus lowest quintiles of the scores, greater adherence to PDI and hPDI was associated with a 19% (Table 2, HR = 0.81, 95% CI: 0.76–0.86) and 17% (HR = 0.83, 95% CI: 0.75–0.92) lower risk of CVD mortality, respectively. In contrast, an increased risk of CVD mortality was observed for uPDI (HR = 1.19, 95% CI: 1.07–1.32). A higher PDI was also associated with a lower risk of death from cancer (HR = 0.86, 95% CI: 0.77–0.96). However, the associations of cancer mortality with hPDI and uPDI were not statistically significant (Table 2).

**Table 2:** Associations between plant-based diet pattern and all-cause and cause-specific mortality risk.

Variable	Included studies	Pooled RR (95% CI)	Included studies	Pooled RR (95% CI)	Included studies	Pooled RR (95% CI)
	All-cause		CVD		Cancer	
PDI	10	0.85 (0.80–0.90)	7	0.81 (0.76–0.86)	6	0.86 (0.77–0.96)
hPDI	13	0.86 (0.81–0.92)	8	0.83 (0.75–0.92)	6	0.89 (0.78–1.01)
uPDI	11	1.20 (1.11–1.31)	8	1.19 (1.07–1.32)	6	1.13 (0.98–1.30)

CVD, cardiovascular disease; RR, relative risk; CI, confidence interval; PDI, plant-based diet index; hPDI, healthful plant-based diet index; uPDI, unhealthful plant-based diet index.

### Additional analyses

**Figure 3:**
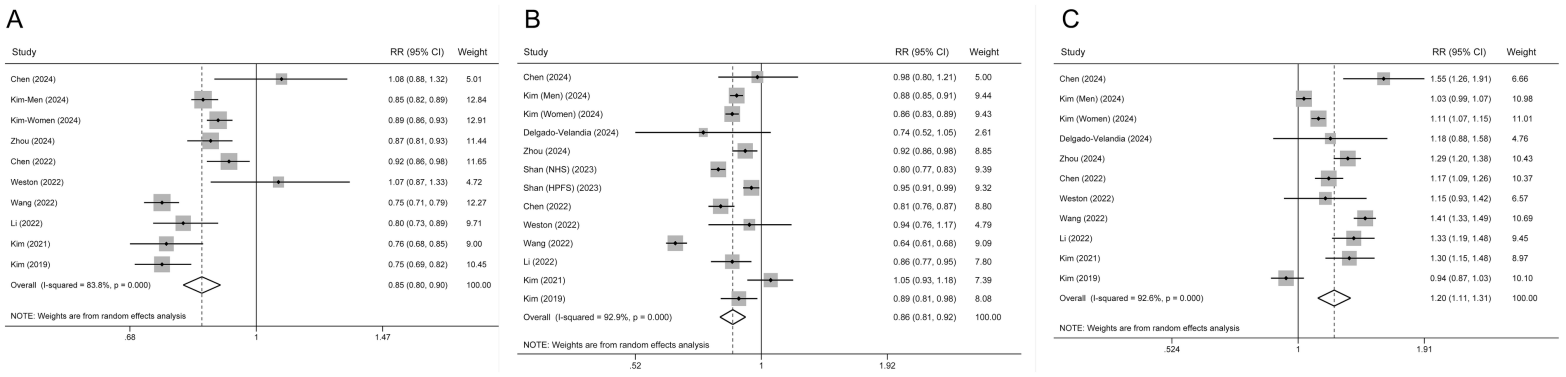
Dose–response associations between PDI **(A)**, hPDI **(B)** and uPDI **(C)** and relative risk for all-cause mortality. Red solid line and green dash lines represent point estimates and 95% confidence intervals for non-linear analysis; blue dash line represents point estimates for linear analysis.

We performed subgroup analyses for all-cause mortality based on geographical region (Europe vs. North America vs. Asia), number of participants (≥50,000 vs. <50,000), and duration of follow-up (≥10 years vs. <10 years). No significant interactions were observed for these factors in most analyses based on meta-regression models, except for follow-up year in uPDI analysis (Table 3). Sensitivity analysis was performed by ruling out each study in turn and repeating the meta-analysis for all-cause mortality. The association remained statistically significant after omitting any individual studies (Supplementary Figure S1). There was no obvious evidence of publication bias as shown in Begg’s funnel plot (Supplementary Figure S2). Dose–response meta-analysis showed that there was a non-linear relationship between PDI or hPDI level and all-cause mortality (Figure 3, *p* = 0.001 and *p* < 0.001 for non-linearity, respectively). No evidence of a non-linear relationship was observed for uPDI (*p* = 0.596 for non-linearity).

**Table 3:** Subgroup analyses of the association between plant-based diet pattern and all-cause mortality risk.

Subgroup	Included studies	Pooled RR (95% CI)	*P*	*P* for interaction	Heterogeneity		
					*Q*	*I*^2^ (%)	*P*
PDI							
Geographical region				0.421			
Asia	3	0.90 (0.76–1.06)	0.194		12.18	83.6	0.002
Europe	1	0.87 (0.81–0.93)	<0.001		–	–	–
Americas	6	0.83 (0.77–0.89)	<0.001		38.38	87.0	<0.001
No. of Participants				0.379			
≥50,000	5	0.83 (0.77–0.88)	<0.001		30.07	86.7	<0.001
<50,000	5	0.89 (0.79–1.01)	0.068		24.72	83.8	<0.001
Follow-up, y				0.800			
≥10	5	0.84 (0.78–0.90)	<0.001		21.54	81.4	<0.001
<10	5	0.86 (0.78–0.95)	0.004		32.18	87.6	<0.001
hPDI							
Geographical region				0.293			
Asia	3	0.93 (0.77–1.13)	0.483		15.15	86.8	0.001
Europe	2	0.89 (0.75–1.04)	0.142		1.43	29.9	0.232
Americas	8	0.84 (0.77–0.91)	<0.001		146.92	95.2	<0.001
No. of participants				0.530			
≥50,000	6	0.84 (0.76–0.93)	0.001		131.45	96.2	<0.001
<50,000	7	0.89 (0.83–0.95)	0.001		18.39	67.4	0.005
Follow-up, y				0.257			
≥10	8	0.89 (0.84–0.94)	<0.001		47.87	85.4	<0.001
<10	5	0.83 (0.70–0.97)	0.022		84.54	95.3	<0.001
uPDI							
Geographical region				0.228			
Asia	3	1.30 (1.13–1.49)	<0.001		7.31	72.6	0.026
Europe	2	1.28 (1.20–1.37)	<0.001		0.34	0.0	0.561
Americas	6	1.15 (1.02–1.29)	0.020		107.38	95.3	<0.001
No. of participants				0.834			
≥50,000	5	1.19 (1.04–1.38)	0.001		98.02	95.9	<0.001
<50,000	6	1.22 (1.08–1.37)	0.014		36.66	86.4	<0.001
Follow-up, y				0.016			
≥10	6	1.09 (1.01–1.17)	0.031		26.75	81.3	<0.001
<10	5	1.32 (1.22–1.44)	<0.001		18.51	78.4	0.001

RR, relative risk; CI, confidence interval; PDI, plant-based diet index; hPDI, healthful plant-based diet index; uPDI, unhealthful plant-based diet index; No., number; y, year.

## Discussion

This comprehensive meta-analysis of cohort studies showed that higher adherence to a PDI and hPDI dietary pattern was linked to a reduced risk of overall mortality. In contrast, greater adherence to an uPDI was associated with an increased risk of all-cause mortality. A similar pattern emerged for CVD mortality. Our findings suggest that a plant-based diet-low in animal foods, sugary drinks, refined grains, and fruit juices-correlates with lower risks of both all-cause and CVD mortality. To our knowledge, this is the first meta-analysis to evaluate the impact of PDIs on mortality risk.

Our study strengthens the evidence supporting a beneficial link between plant-based diets, including healthful plant-based foods, and reduced mortality. We also highlight the increased risk of mortality associated with frequent consumption of unhealthful plant-based foods. A plant-based diet refers to an eating pattern that prioritizes foods derived from plants. It can vary in its degree of restriction and may not necessarily exclude animal products completely. Healthful plant-based foods refer specifically to the quality and nutritional value of the foods within the plant-based spectrum. Our findings emphasize that the quality of plant foods is just as important as the quantity of plant-based foods.

Several mechanisms may explain the potential associations between adherence to a plant-based diet or a healthy plant-based pattern and lower risk of mortality. Diets emphasizing whole plant foods, including fruits, vegetables, legumes, whole grains, nuts, and seeds, are rich in essential nutrients, antioxidants, and dietary fiber, which collectively contribute to a lower risk of chronic diseases such as CVD, diabetes, and certain cancers (32–34). Moreover, plant-based diets are associated with improved metabolic markers, reduced inflammatory markers, and healthier lipid profiles, all of which are critical in reducing the risk of premature death (35–37). On the other hand, diets low in plant-based components and high in animal products, particularly red and processed meats, have been correlated with higher mortality risk (38, 39).

A non-significant association between both the hPDI and uPDI and cancer mortality was observed in our study. This was unexpected, as previous research has linked high fiber intake and reduced consumption of red and processed meats with a lower risk of cancer mortality (40, 41). One explanation for this null association could be inaccuracies in the cause-of-death information on death certificates, especially concerning cancer. Another possibility is that participants diagnosed with cancer may have changed their dietary habits after their diagnosis. Furthermore, the presence of associations may vary depending on the specific type of cancer mortality being studied (42).

Our meta-analysis has several strengths. First, it included a large sample size and had substantial statistical power, with only prospective studies considered. Second, the methodological quality of the included studies was generally high, as assessed by the NOS. Third, we conducted both categorical and dose–response analyses, which enhanced the robustness and reliability of the results. Lastly, the PDI scores in each study were calculated using a consistent methodology, enhancing the comparability of the studies.

However, some limitations should be noted. First, while no significant publication bias was detected through Begg’s and Egger’s tests, there is still the possibility of bias, as smaller studies with null results may be less likely to be published. Second, variations in dietary assessment methods and cut-off points across the included studies could have affected the pooled results. Third, significant heterogeneity among the studies may reduce the overall strength of our conclusions. Lastly, meta-analyses may not fully address confounding factors that are present in the studies they include. The potential for unmeasured or residual confounding remains, which could influence the outcomes and limit the ability to draw definitive conclusions.

In conclusion, greater adherence to a PDI or hPDI dietary pattern was associated with a lower risk of all-cause and CVD mortality, whereas uPDI dietary pattern was negatively associated with mortality risk. Thus, promoting a plant-based dietary pattern may be a key strategy in improving public health and reducing the burden of diet-related mortality.

## Data Availability

The raw data supporting the conclusions of this article will be made available by the authors, without undue reservation.

## Footnotes

^1^ http://www.ohri.ca/programs/clinical_epidemiology/oxford.asp

## Supplementary material

SUPPLEMENTARY FIGURE S1 Sensitivity analysis was performed whereby each study was omitted in turn and the pooled risk estimates were recalculated to determine the influence of each study. **(A)** PDI, **(B)** hPDI and **(C)** uPDI.

SUPPLEMENTARY FIGURE S2 Evaluation of publication bias with a Begg’s funnel plot. **(A)** PDI, **(B)** hPDI and **(C)** uPDI.
