# Supplementary material for: Plant-based diets and total and cause-specific mortality: a meta-analysis of prospective studies
Source: Front Nutr. 2025 Jan 20;12:1518519. doi: 10.3389/fnut.2025.1518519 (PMC11788165; doi:10.3389/fnut.2025.1518519)
Supplement: Supplementary file 1 [file Table_1.docx]

**Table S1**. Methodological quality of studies included in the meta-analysis

| Cohort study^1^ | Representativeness of the exposed  cohort | Selection of the  unexposed  cohort | Ascertainment  of exposure | Outcome not  present at  start of study | Comparability ^2^ | Outcome  assessment | Follow-up long enough ^3^ | Adequacy  of follow-up ^4^ | Total  quality  scores |
| --- | --- | --- | --- | --- | --- | --- | --- | --- | --- |
| Chen et al., 2024 | ☆ | ☆ | - | ☆ | ☆☆ | ☆ | - | ☆ | 7 |
| Kim-Men, 2024 | ☆ | ☆ | - | ☆ | ☆☆ | ☆ | ☆ | ☆ | 8 |
| Kim-Women, 2024 | ☆ | ☆ | - | ☆ | ☆☆ | ☆ | ☆ | ☆ | 8 |
| Delgado-Velandia et al., 2024 | ☆ | ☆ | - | ☆ | ☆☆ | ☆ | ☆ | ☆ | 8 |
| Zhou et al., 2024 | ☆ | ☆ | - | ☆ | ☆☆ | ☆ | - | ☆ | 7 |
| Shan et al., 2023-HNS | ☆ | ☆ | - | ☆ | ☆☆ | ☆ | ☆ | ☆ | 8 |
| Shan et al., 2023-HPFS | ☆ | ☆ | - | ☆ | ☆☆ | ☆ | ☆ | ☆ | 8 |
| Chen et al., 2022 | ☆ | ☆ | - | ☆ | ☆☆ | ☆ | - | ☆ | 7 |
| Weston et al., 2022 | ☆ | ☆ | ☆ | ☆ | ☆☆ | ☆ | ☆ | ☆ | 9 |
| Wang et al., 2022 | ☆ | ☆ | - | ☆ | ☆☆ | ☆ | - | ☆ | 7 |
| Li et al., 2022 | ☆ | ☆ | ☆ | ☆ | ☆☆ | ☆ | - | ☆ | 8 |
| Kim et al., 2021 | ☆ | ☆ | - | ☆ | ☆☆ | ☆ | ☆ | ☆ | 8 |
| Kim et al., 2019 | ☆ | ☆ | ☆ | ☆ | ☆☆ | ☆ | ☆ | ☆ | 9 |

^1^ A study can be awarded a maximum of one star for each item except the item ‘Comparability’.

^2^ A maximum of two stars can be awarded for this item. Studies controlling for age received one star while studies additionally controlling for other important confounders received an additional star.

^3^ A cohort study with a follow-up time more than 10 years was assigned one star.

^4^ A cohort study with a follow-up rate more than 75% was assigned one star.
