# Supplementary figures and images for: Plant-based diets and total and cause-specific mortality: a meta-analysis of prospective studies
Source: Front Nutr. 2025 Jan 20;12:1518519. doi: 10.3389/fnut.2025.1518519 (PMC11788165; doi:10.3389/fnut.2025.1518519)

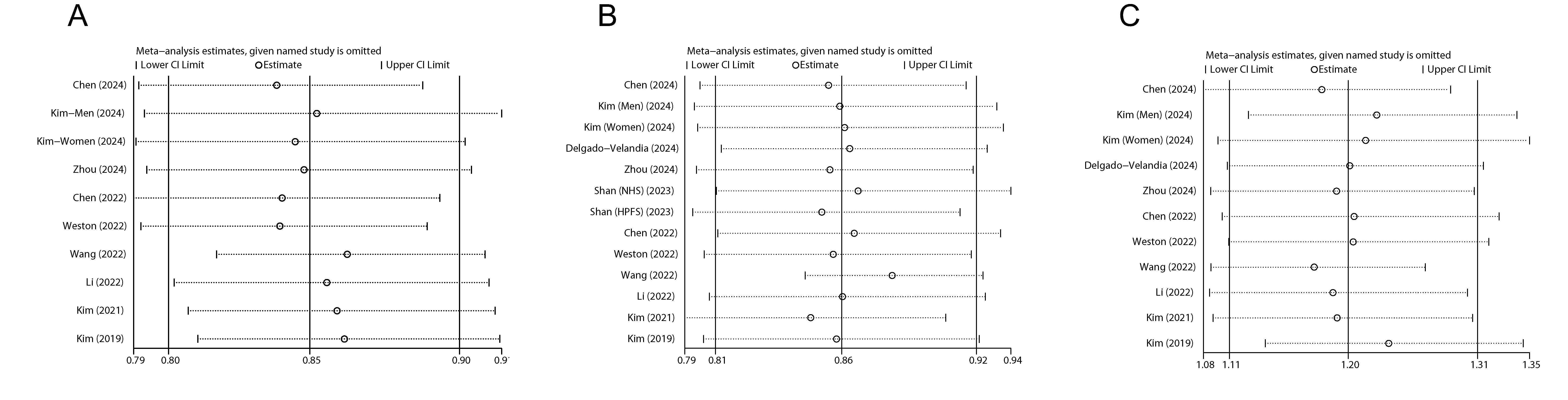

Supplement: SUPPLEMENTARY FIGURE S1 — Sensitivity analysis was performed whereby each study was omitted in turn and the pooled risk estimates were recalculated to determine the influence of each study. (A) PDI, (B) hPDI and (C) uPDI. [file Image_1.tif]

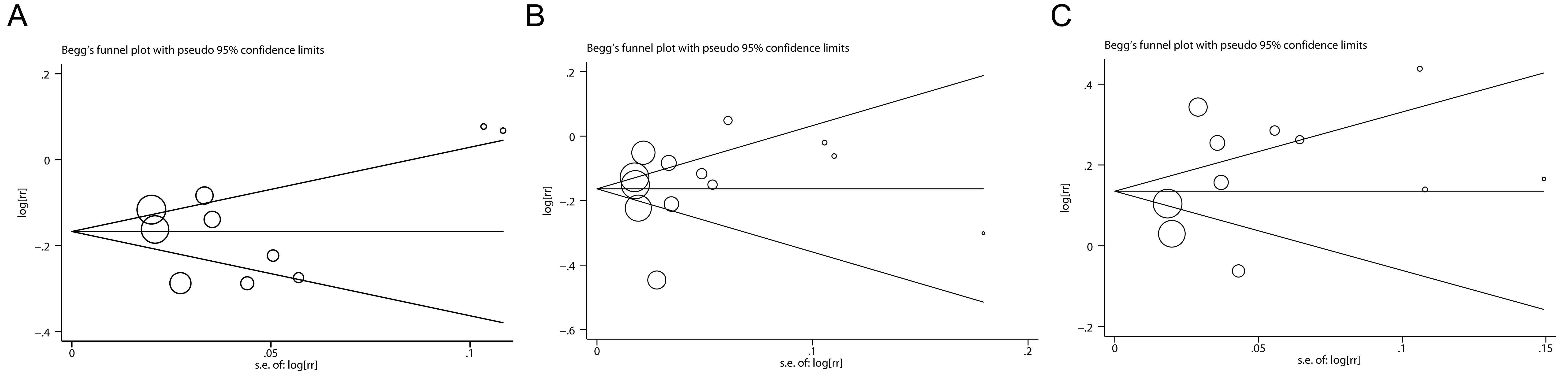

Supplement: SUPPLEMENTARY FIGURE S2 — Evaluation of publication bias with a Begg’s funnel plot. (A) PDI, (B) hPDI and (C) uPDI. [file Image_2.tif]
